# Evaluation of the performance of a novel chemiluminescence immunoassay for cryptococcal antigen detection

**DOI:** 10.1128/spectrum.03545-25

**Published:** 2026-03-10

**Authors:** Ying Zhao, Lijiang Chen, Dingding Li, Xiurong Ding, Yunsong Yu, Zhonghao Wang, Yingchun Xu, Lina Guo

**Affiliations:** 1 Department of Laboratory Medicine, State Key Laboratory of Complex, Severe, and Rare Diseases, Peking Union Medical College Hospital, Chinese Academy of Medical Sciences and Peking Union Medical College 34732, Beijing, People’s Republic of China; 2 Graduate School, Chinese Academy of Medical Sciences and Peking Union Medical College 12501, Beijing, People’s Republic of China; 3 Department of Clinical Laboratory, Key Laboratory of Clinical Laboratory Diagnosis and Translational Research of Zhejiang Province, The First Affiliated Hospital of Wenzhou Medical University 89657, Wenzhou, Zhejiang, People’s Republic of China; 4 Department of Clinical Laboratory, Beijing You'an Hospital, Capital Medical University 12517, Beijing, People’s Republic of China; 5 Department of Infectious Diseases, Sir Run Run Shaw Hospital affiliated with Zhejiang University School of Medicine 74678, Hangzhou, Zhejiang, People’s Republic of China; 6 Department of Laboratory Medicine, West China Hospital of Sichuan University 34753, Chengdu, Sichuan, People’s Republic of China; MultiCare Health System, Tacoma, Washington, USA

**Keywords:** cryptococcosis, glucuronoxylomannan antigen, chemiluminescence immunoassay, lateral flow assay

## Abstract

This study evaluated the diagnostic performance of a chemiluminescence immunoassay (CLIA) for detecting the *Cryptococcus* glucuronoxylomannan (GXM) antigen and assessed its agreement with the U.S. Food and Drug Administration (FDA)-approved IMMY lateral flow assay (LFA). From June 2021 to September 2023, a total of 651 specimens (633 cerebrospinal fluid, 18 serum) collected from 208 cryptococcosis and 415 non-cryptococcosis patients (based on diagnostic criteria for cryptococcosis) were analyzed by Dynamiker GXM antigen-CLIA platform and IMMY GXM antigen-LFA platform. Among 623 participants, the CLIA method demonstrated a total percentage agreement (TPA) of 99.52% relative to LFA. Sensitivity, specificity, positive predictive value (PPV), and negative predictive value (NPV) for CLIA were 99.04, 100, 100, and 99.52%, respectively. Serial monitoring in patients receiving antifungal therapy showed a progressive decline in GXM antigen concentrations over time. The CLIA-based GXM assay exhibited high sensitivity and specificity for the diagnosing of cryptococcosis and showed close alignment with the FDA-approved LFA. It enables tracking antigen concentration changes during treatment, providing preliminary reference for therapeutic monitoring.

**IMPORTANCE:**

This study demonstrates that the novel chemiluminescence immunoassay (CLIA) and the conventionally used LFA have a comparable performance in the detection of glucuronoxylomannan in cerebrospinal fluid (CSF) among patients with cryptococcosis. It enables tracking antigen concentration changes during treatment, providing preliminary reference for therapeutic monitoring.

## INTRODUCTION

**Fig 1:**
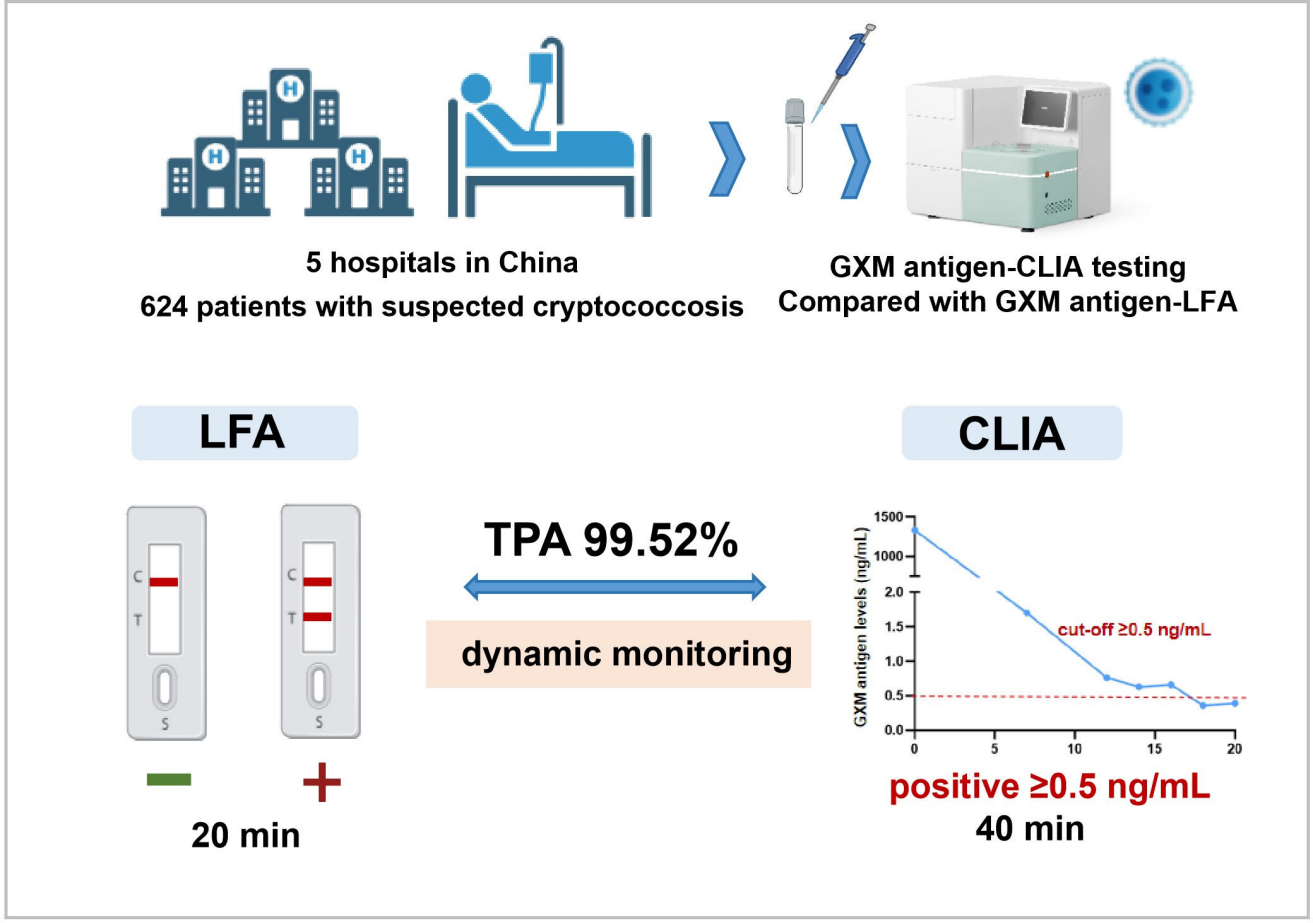
Schematic diagram of the study.

Cryptococcosis is a subacute-to-chronic invasive mycosis caused by *Cryptococcus* species, with the central nervous system as the most frequent site of infection, followed by pulmonary and cutaneous involvement (1, 2). Infections caused by *Cryptococcus neoformans* and *Cryptococcus gattii* account for the majority of human disease (3). Although these fungi are classically opportunistic pathogens affecting immunocompromised hosts, cases in immunocompetent individuals have been documented. According to World Health Organization (WHO) estimates, cryptococcal meningitis contributes to roughly 19% of global AIDS-related mortality, with an annual incidence exceeding 180,000 cases concentrated in sub-Saharan Africa and Southeast Asia (4). High-risk populations include individuals with advanced HIV infection (CD4 count < 100 cells/µL), recipients of solid organ transplants, patients on prolonged corticosteroid therapy, and those with hematologic malignancies (5). Severe clinical presentations often involve meningitis or disseminated infection, with mortality rates remaining above 40%, even when antifungal agents are available, underscoring the importance of rapid diagnosis and treatment initiation. Conventional diagnostic strategies, such as India ink microscopy, culture isolation, and cryptococcal antigen testing, each present limitations (6). Culture remains the definitive diagnostic method but is slow (often 3–5 days) (7); India ink staining is rapid, but its sensitivity is operator-dependent, ranging from 50 to 80% (8–11); and lateral flow assays (LFAs) are inexpensive, easy to use, and widely adopted, but their qualitative/semi-quantitative readout limits their role in monitoring disease progression or therapeutic response. Although the IMMY LFA provides semi-quantitative results, it relies on a cumbersome manual dilution process that yields only an approximate concentration range and fails to reflect precise antigen dynamics. While metagenomic next-generation sequencing (mNGS) offers broad pathogen detection, its high cost restricts routine use (12). Chemiluminescence immunoassays (CLIAs) have gained attention for their combination of speed, affordability, high-throughput capacity, and clinically relevant sensitivity, alongside the added benefit of quantitative measurement (13, 14). In this work, we investigated the diagnostic performance of glucuronoxylomannan (GXM) antigen-CLIA and assessed its agreement with the IMMY LFA (Fig. 1).

## MATERIALS AND METHODS

### Study design

This was a multicenter case-control study involving five major hospitals: Peking Union Medical College Hospital, Beijing You’an Hospital, Capital Medical University, The First Affiliated Hospital of Wenzhou Medical University, Sir Run Run Shaw Hospital, Zhejiang University School of Medicine, and West China Hospital of Sichuan University. Between June 2021 and September 2023, a total of 624 patients with suspected cryptococcosis were included. The exclusion criteria were as follows: (i) remaining samples with insufficient amount and (ii) incomplete medical records. After excluding one case, diagnoses were established according to the 2019 European Organization for Research and Treatment of Cancer (EORTC)/Mycoses Study Group Education and Research Consortium (MSGERC) consensus criteria (15) and the Chinese Expert Consensus on the Diagnosis and Treatment of Cryptococcal Meningitis (16). A total of 208 cases were confirmed as cryptococcosis based on the following diagnostic criteria: (i) identification of *Cryptococcus neoformans* or *Cryptococcus gattii* in sterile-site specimens (serum, CSF, or lung tissue) via India ink staining or culture; (ii) pathological detection of *Cryptococcus* in lung tissue biopsy specimens; (iii) cryptococcal antigen positivity in serum or CSF; and (iv) patients with cryptococcosis were classified into pulmonary cryptococcosis, cryptococcal meningitis, and disseminated cryptococcosis by two experienced clinicians based on clinical manifestations.

### Testing approach

The CSF samples used in this study were residual from routine clinical testing, which included both fresh and frozen samples. All CSF samples underwent simultaneous GXM antigen-CLIA and GXM antigen-LFA testing. Fresh CSF samples (*n* = 115) were tested within 4 h of collection. Frozen CSF samples (*n* = 508) were stored at −20°C immediately after routine clinical testing. Before analysis, they were thawed at room temperature (10–30°C) and gently mixed to ensure homogeneity. Serum samples were residuals stored at −20°C. After thawing at room temperature, they were tested via GXM antigen-CLIA.

The CLIA used was a streptavidin-coated magnetic particle-based *Cryptococcus* GXM Antigen Detection Kit (Dynamiker, Tianjin, China). For each test, 100 μL of specimen or control was added to the reagent strip. Samples were first incubated at 37°C for 8 min with a biotinylated anti-GXM capture antibody and a sulfonamide-acridinium ester-conjugated detection antibody. Streptavidin-coated magnetic beads were subsequently introduced, followed by another 8-min incubation at 37°C. After washing to remove excess reagents, immunocomplexes consisting of bead-bound capture antibody, GXM antigen, and acridinium-labeled detection antibody were formed in positive samples. Upon addition of the trigger solution, chemiluminescent signals were recorded as relative luminescent units (RLUs) directly proportional to GXM concentration. A cutoff of ≥0.5 ng/mL was defined as positive, indicating likely cryptococcal infection.

For LFA, we used the IMMY “sandwich” immunochromatographic test strip for cryptococcal antigen (CrAg) detection. A positive test resulted in two lines (the control and test lines); a negative test resulted in only one line (the control line); and if the band did not appear, the test was invalid.

### Statistics

Continuous variables with normal distributions were expressed as the mean ± standard deviation (χ ± s) and compared using independent-sample *t*-tests. Non-normally distributed data were presented as medians (interquartile range, Q25–Q75) and analyzed with the Mann-Whitney *U* test. Categorical variables are reported as counts (percentages). Sensitivity, specificity, PPV, NPV, and TPA were calculated for both CLIA and LFA. Receiver operating characteristic (ROC) curves were constructed, and the area under the curve (AUC) values were compared to assess diagnostic performance. Statistical analyses were performed in GraphPad Prism 10.1.2 with a significance threshold of *P* < 0.05 (two-tailed).

## RESULTS

### Patient clinical data

A total of 651 specimens (633 CSF, 18 serum) from 623 patients were analyzed, including 208 with cryptococcosis and 415 controls. Serum samples were reserved for antifungal treatment monitoring. Table 1 summarizes demographic and clinical characteristics. Cryptococcosis patients were generally older and more often male than controls (*P* < 0.05). Organ transplantation was significantly more frequent in the cryptococcosis cohort (*P* < 0.01). Common presenting features included intracranial hypertension, cranial nerve deficits, and brain parenchymal lesions, whereas fever and respiratory tract symptoms were relatively uncommon.

**TABLE 1:** Characteristics of cryptococcosis and non-cryptococcosis patients^*a*^

Clinical feature	Cryptococcosis (*n* = 208)	Non-cryptococcosis (*n* = 415)	*P*-value
Age, years (median [IQR])	50 [37, 62]	43 [29, 58]	0.0002
Male, *n* (%)	147 (70.7)	252 (60.7)	0.0147
Immunodeficiency, *n* (%)	128 (61.5)	200 (48.2)	0.0017
Underlying disease, *n* (%)			
HIV	53 (25.5)	84 (20.2)	0.1364
Organ transplantation	14 (6.7)	4 (1.0)	0.0001
Blood cancers/malignant tumors	9 (4.3)	22 (5.3)	0.5979
Autoimmune diseases	13 (6.3)	27 (6.5)	0.9021
Hypertension	21 (10.1)	51 (12.3)	0.4194
Diabetes	34 (16.4)	51 (12.3)	0.1642
Pulmonary diseases	33 (15.9)	48 (11.6)	0.1324
Clinical findings, *n* (%)			
Intracranial hypertension	148 (63.4)	231 (56.5)	<0.0001
Cranial neuropathies	3 (1.4)	26 (6.3)	0.0075
Parenchymal brain lesions	7 (3.4)	40 (9.6)	0.0052
Fever/respiratory tract infection	35 (16.8)	75 (18.1)	0.7006
GXM antigen levels, ng/mL (median [IQR])	30.8 [1.90, 100]	0 [0, 0.01]	<0.0001

^*a*^ HIV, human immunodeficiency virus; IQR, interquartile range.

### Comparison of CLIA and LFA consistency

A total of 623 samples were tested in parallel using both GXM-LFA and GXM-CLIA assays. Of the 207 samples that tested positive by GXM-LFA, 205 were also positive by GXM-CLIA, yielding a positive percentage agreement (PPA) of 99.03%. Among the 416 samples negative by GXM-LFA, 415 were negative by GXM-CLIA, resulting in a negative percentage agreement (NPA) of 99.76%. The overall agreement between the two assays was 99.52%. Cohen’s kappa was 0.9891 (>0.75), indicating almost perfect inter-assay reliability (Table 2). Discrepancies were limited to three specimens, all from patients with confirmed cryptococcal disease. Two were LFA-positive but CLIA-negative, while one was CLIA-positive but LFA-negative (Table 3).

**TABLE 2:** Observed detection agreement between CLIA and LFA approaches^*a*^

Sample type	PPA, % (95% CI)	NPA, % (95% CI)	TPA, % (95% CI)	Kappa
CFS	99.03 (205/207) (96.55–99.73)	99.76 (415/416) (98.65–99.96)	99.52 (620/623) (98.59–99.84)	0.9891

^*a*^ PPA, positive percent agreement; NPA, negative percent agreement; TPA, total percent agreement.

**TABLE 3:** Discordant results observed for three samples

Sample	Age	Gender	Clinical diagnosis	LFA result	CLIA result (ng/mL)
1	39	Female	Cryptococcal meningitis	Negative (−)	0.67
2	58	Male	Cryptococcal meningitis	Positive (+)	0.01
3	34	Female	Cryptococcal meningitis	Positive (+)	0.00

### GXM antigen CLIA performance

**Fig 2:**
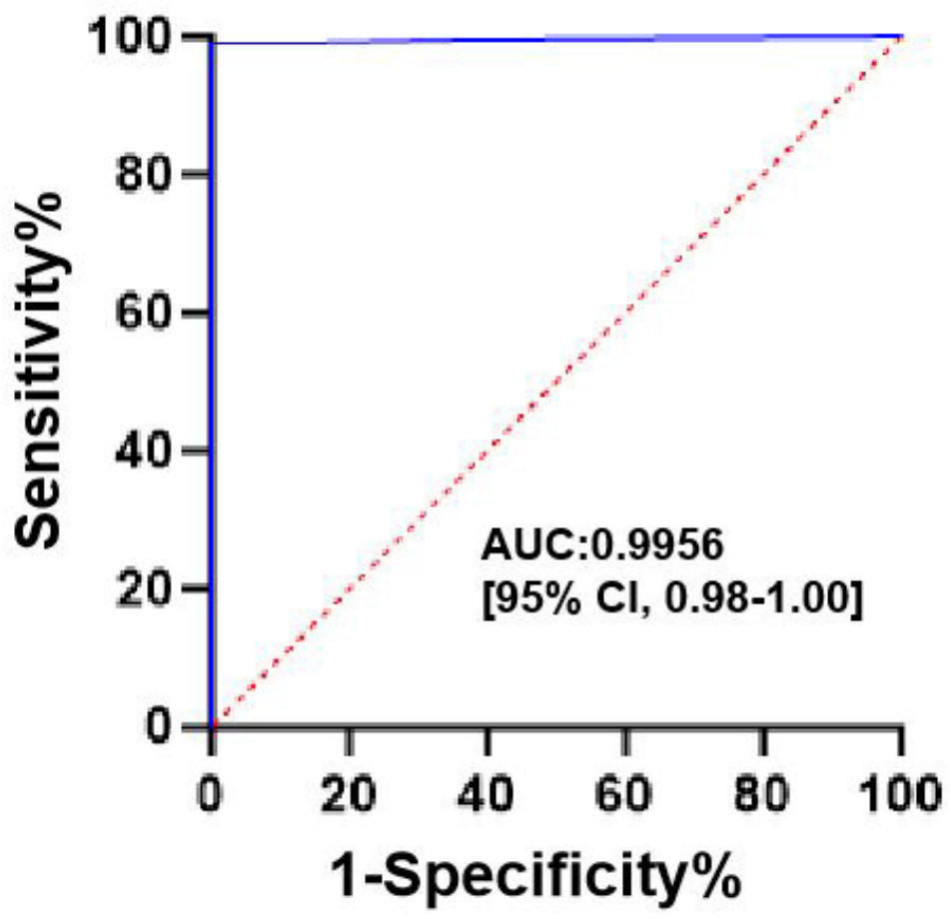
ROC curve analysis.

When applying the manufacturer’s recommended threshold of 0.5 ng/mL for GXM antigen positivity, the CLIA achieved a sensitivity of 99.04% and a specificity of 100%, while its specificity matched the LFA’s (100%), and its sensitivity was slightly lower than the LFA’s (99.52%). (Table 4) ROC curve analysis showed outstanding diagnostic performance, with an AUC of 0.9956 (95% confidence interval [CI]: 0.9885–1.000; Fig. 2). At the 0.5 ng/mL cutoff, the Youden index reached its maximum value of 0.9904, corresponding to the aforementioned sensitivity and specificity values.

**TABLE 4:** Performance parameters for the GXM antigen-based CLIA

Performance parameters	GXM antigen-CLIA	GXM antigen-LFA
Sensitivity, % (95% CI)	99.04 (206/208) (96.20–99.83)	99.52 (207/208) (96.93–99.97)
Specificity, % (95% CI)	100 (415/415) (98.86–100)	100 (415/415) (98.6–100)
PPV, % (95% CI)	100 (206/206) (97.72–100)	100 (207/207) (97.72–100)
NPV, % (95% CI)	99.52 (415/417) (98.09–99.92)	99.76 (414/415) (98.45–99.99)

### Analysis of detection levels

**Fig 3:**
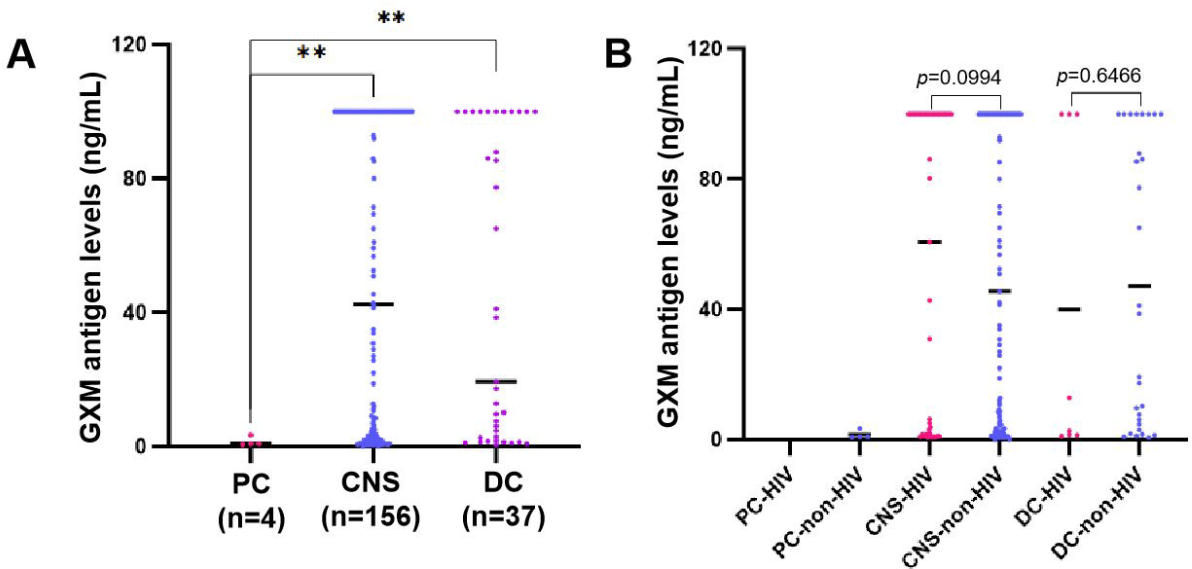
Levels of GXM antigen detection. (**A**) GXM antigen levels in different cryptococcal diseases. (**B**) Comparative GXM antigen levels in HIV vs. non-HIV cryptococcal disease. **P＜0.01. PC, pulmonary cryptococcosis; CNS, central nervous system cryptococcosis; DC, disseminated cryptococcosis; and HIV, human immunodeficiency virus.

The 208 confirmed cryptococcosis cases were stratified into four categories based on clinical presentation: pulmonary cryptococcosis (PC, *n* = 4), central nervous system cryptococcosis (CNS, *n* = 156), disseminated cryptococcosis (DC, *n* = 37), and other manifestations (*n* = 11). Compared with the non-cryptococcosis cohort, GXM antigen concentrations were markedly elevated in all cryptococcosis groups (*P* < 0.01; Table 1). Among cerebrospinal fluid (CSF) samples, antigen levels were highest in CNS disease (49.91 ± 3.60 ng/mL), followed by DC (45.56 ± 7.26 ng/mL) and PC (1.42 ± 0.68 ng/mL). Both CNS and DC groups had significantly greater antigen burdens than PC (*P* < 0.01). None of the PC cases were HIV-positive. Within the CNS group, HIV-coinfected individuals exhibited higher mean antigen concentrations than HIV-negative patients, though the difference did not reach statistical significance (*P* > 0.05; Fig. 3).

### Prognostic assessment of GXM antigen levels

**Fig 4:**
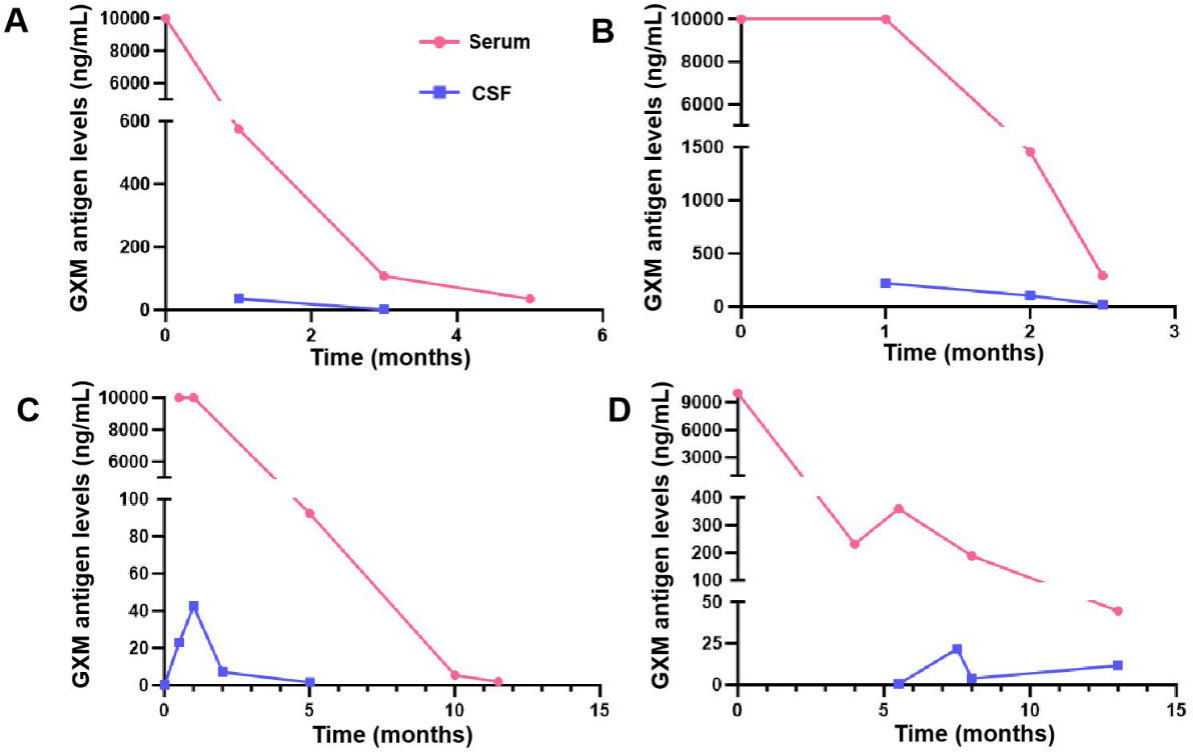
GXM antigen dynamics in serum and CSF from four patients. (**A**) Patient 1, (**B**) patient 2, (**C**) patient 3, and (**D**) patient 4. Red lines: serum; blue lines: CSF (ng/mL).

Serial monitoring data were available for four patients, comprising 32 samples in total (18 serum, 14 CSF). In all cases, serum antigen concentrations exceeded those measured in the CSF. Across the treatment course, each patient demonstrated a steady reduction in antigen levels, consistent with microbiological clearance during antifungal therapy (Fig. 4).

## DISCUSSION

Cryptococcosis remains a major cause of morbidity and mortality worldwide. Detection of the GXM antigen in serum or CSF is widely recognized as a cornerstone of early diagnosis and treatment initiation (14, 17, 18). Although LFAs are simple, rapid, and cost-effective, quantifying antigen titers via LFA requires multiple test strips, sufficient reagent supply, and trained personnel. Moreover, LFA yields only semi-quantitative results (19, 20). The CLIA technology addresses these shortcomings by providing rapid, reproducible, and quantitative measurements (21).

In the present work, the Dynamiker GXM antigen CLIA was benchmarked against the U.S. FDA-approved IMMY LFA. The two methods demonstrated almost perfect agreement, with an overall concordance rate of 99.52%. To investigate potential cross-reactivity, 67 samples from patients with other neuroinflammatory diseases, including viral meningitis (*n* = 27), tuberculous meningitis/encephalitis (*n* = 21), autoimmune encephalitis/encephalopathy (*n* = 13), bacterial meningitis (*n* = 5), and eosinophilic meningoencephalitis (*n* = 1), were evaluated, with all testing negative for GXM antigen.

Using the manufacturer-recommended 0.5 ng/mL threshold as a cutoff, CLIA achieved 99.04% sensitivity and 100% specificity for cryptococcosis diagnosis in the CSF, in line with prior work. For example, Li et al. reported CLIA sensitivity of 100% and specificity of 97.67% at a cutoff of 2 ng/m (22), while Li et al. documented 100% sensitivity and specificity (23).

Our results also reinforce the association between CSF antigen burden and disease severity. Patients with CNS involvement had markedly higher GXM antigen levels than those with isolated pulmonary disease (*P* < 0.01), mirroring prior findings linking elevated serum antigen titers to adverse outcomes. Hu et al., for instance, showed that serum titers ≥1:320 independently predicted cryptococcal meningitis (24).

Beyond diagnosis, antigen testing offers value for treatment monitoring. Several studies have shown that changes in GXM titers can reflect therapeutic response (25–28). In our longitudinal subset, antigen concentrations consistently declined during antifungal therapy, suggesting utility as a biomarker for clearance. Unlike LFA strategies, which may yield unchanged titers despite gradual fungal eradication (22, 29), CLIA approaches provide concentration values, allowing for sensitive detection of even modest antigen reductions.

This work has several factors that limit its interpretation. First, the diagnostic performance of serum-based CLIA was not systematically assessed. However, serum is more accessible in clinical practice. Thus, combining paired CSF and serum samples to compare the assay’s performance across sample types would be critical for guiding its utility in diverse clinical settings. Second, follow-up data for therapeutic monitoring were limited, restricting our ability to determine the time to antigen negativity. Moreover, due to the limited availability of longitudinal monitoring data sets, the potential value of GXM antigen for therapeutic monitoring requires validation in large-scale, prospective, long-term follow-up cohorts. Third, *Cryptococcus neoformans* and *Cryptococcus gattii* differ in pathogenicity, geography, and clinical presentation. Without data on the causative species, we could not assess assay performance differences between them, which is critical for diagnostic and clinical management, including optimizing antifungal therapy and duration. Fourth, the diagnostic performance of GXM antigen-CLIA was not compared against fungal culture, the traditional gold standard for invasive fungal disease diagnosis. We adopted a composite reference standard to improve case identification accuracy. This design reduces the direct comparability of our findings with those of similar studies based on the gold standard.

In conclusion, this multicenter evaluation demonstrates that the GXM antigen CLIA delivers high sensitivity and specificity and near-complete agreement with an FDA-cleared LFA. Given its ability to track antigen concentration changes, CLIA may serve as a valuable adjunct for tracking cryptococcal clearance during antifungal therapy.

## Data Availability

The authors confirm that the data supporting the findings of this study are available within the article.
